# Temporal Variability of Urinary Phthalate Metabolite Levels in Men of Reproductive Age

**DOI:** 10.1289/ehp.7212

**Published:** 2004-08-16

**Authors:** Russ Hauser, John D. Meeker, Sohee Park, Manori J. Silva, Antonia M. Calafat

**Affiliations:** ^1^Department of Environmental Health, Harvard School of Public Health, Boston, Massachusetts, USA; ^2^Vincent Memorial Obstetrics and Gynecology Service, Andrology Laboratory and In Vitro Fertilization Unit, Massachusetts General Hospital, Boston, Massachusetts, USA; ^3^Department of Biostatistics, Harvard School of Public Health, Boston, Massachusetts, USA; ^4^National Center for Environmental Health, Centers for Disease Control and Prevention, Atlanta, Georgia, USA

**Keywords:** biomarkers, human, phthalates, reliability, urine

## Abstract

Phthalates are a family of multifunctional chemicals widely used in personal care and other consumer products. The ubiquitous use of phthalates results in human exposure through multiple sources and routes, including dietary ingestion, dermal absorption, inhalation, and parenteral exposure from medical devices containing phthalates. We explored the temporal variability over 3 months in urinary phthalate metabolite levels among 11 men who collected up to nine urine samples each during this time period. Eight phthalate metabolites were measured by solid-phase extraction–high-performance liquid chromatography–tandem mass spectrometry. Statistical analyses were performed to determine the between- and within-subject variance apportionment, and the sensitivity and specificity of a single urine sample to classify a subject’s 3-month average exposure. Five of the eight phthalates were frequently detected. Monoethyl phthalate (MEP) was detected in 100% of samples; monobutyl phthalate, monobenzyl phthalate, mono-2-ethylhexyl phthalate (MEHP), and monomethyl phthalate were detected in > 90% of samples. Although we found both substantial day-to-day and month-to-month variability in each individual’s urinary phthalate metabolite levels, a single urine sample was moderately predictive of each subject’s exposure over 3 months. The sensitivities ranged from 0.56 to 0.74. Both the degree of between- and within-subject variance and the predictive ability of a single urine sample differed among phthalate metabolites. In particular, a single urine sample was most predictive for MEP and least predictive for MEHP. These results suggest that the most efficient exposure assessment strategy for a particular study may depend on the phthalates of interest.

Phthalates, diesters of phthalic acid, are a family of multifunctional chemicals that are widely used in personal and consumer products. Phthalates are used to hold color and scent in consumer and personal care products (Koo et al. 2002); as solvents in paints, glue, insect repellents, lubricants, and adhesives [Agency for Toxic Substances and Disease Registry (ATSDR 2001]; and to soften a wide range of plastics (Bradbury 1996), including polyvinyl chloride (PVC) used in the manufacture of medical products such as blood, intravenous, and dialysate bags and tubing (Nassberger et al. 1987). Diethyl phthalate (DEP), di-*n*-butyl phthalate (DBP), and butyl benzyl phthalate (BBzP) are principally used in personal care products, such as body lotions, gels, shampoos, and deodorants (ATSDR 1995, 2001). They also have U.S. Food and Drug Administration approval for uses in food packaging and processing materials that are in contact with food, and as a result they have been found in food (Castle et al. 1990; Page and Lacroix 1995). DBP, BBzP, and di-(2-ethylhexyl) phthalate are also used in residential building materials such as floorings, paints, carpet backings, adhesives, and wallpaper, and in PVC products such as auto parts and interiors (ATSDR 2001, 2002). Although the volatility of phthalates is relatively low, studies have shown that phthalates are present in residential indoor air (Jaakkoala et al. 1999; Rudel et al. 2003).

The ubiquitous use of phthalates results in human exposure via dietary ingestion of foods (such as milk, butter, and meats), dermal absorption of low-molecular-weight phthalates (e.g., DEP, DBP, BBzP), inhalation of the more volatile phthalates, and parenteral exposure from medical devices containing phthalates (ATSDR 1995, 2001, 2002). Recently, researchers at the Centers for Disease Control and Prevention (CDC) developed analytical methods for the quantitative detection of phthalate metabolites in urine (Blount et al. 2000). Phthalate monoester metabolites were measured because of potential sample contamination from the parent diesters and because the metabolites are considered the biologically active toxicant (Li et al. 1998; Peck and Albro 1982: Sjoberg et al. 1986). The use of phthalate metabolites in urine as biomarkers of exposure now allows researchers to accurately measure human exposure to phthalates. These biomarkers represent an integrative measure of phthalate exposure from multiple sources and pathways. Recently, four phthalate metabolites—monoethyl phthalate (MEP), mono-(2-ethylhexyl) phthalate (MEHP), monobutyl phthalate (MBP), and monobenzyl phthalate (MBzP)—were found in the urine samples of > 75% of approximately 2,550 participants of the National Health Nutrition and Examination Survey (NHANES) 1999–2000 (CDC 2003; Silva et al. 2004).

Because humans and other mammals rapidly metabolize phthalate diesters to their respective monoesters, which in turn may be further metabolized, phthalates do not bio-accumulate (ATSDR 1995, 2001, 2002; Peck and Albro 1982). Because biologic half-lives of phthalates are on the order of hours, urinary metabolite levels reflect exposures that most likely occurred ≤1 day preceding the collection of the urine specimen. However, because most health end points of interest are likely associated with exposures over time intervals longer than a few days, information on the temporal variability of urinary levels of phthalate metabolites is needed to optimize the design of exposure assessment in epidemiologic studies. Currently there are limited published data on the temporal variability of urinary phthalate monoester metabolite concentrations. A recent study documented good reproducibility of urinary phthalate monoester levels in two first-morning urine specimens collected for 2 consecutive days; day-to-day intraclass correlation coefficients (ICCs) ranged from 0.5 to 0.8 (Hoppin et al. 2002). Time intervals beyond a couple of days were not explored.

Variability in an individual’s exposure to phthalates can result from changes in the use of personal care products, diet, or daily activity patterns, such as time spent in specific micro-environments (i.e., residential, workplace, or other) with ambient phthalate levels. Therefore, characterizing an individual’s phthalate exposure is complex, and exposure may vary considerably over short time periods, such as days. Although phthalate bio-markers in urine are available to accurately measure a person’s exposure at a single point in time, determining exposure over time intervals of weeks or months will require multiple measurements of phthalate metabolites. Therefore, the present study was designed to explore the temporal variability in urinary phthalate metabolite levels. Our design allowed us to determine between- and within-subject variability in urinary phthalate metabolite levels, as well as apportion the within-person variability into monthly and daily variances. We also explored the sensitivity of a single urine measurement to predict an individual’s 3-month average exposure. This information can be used for designing exposure assessment strategies for epidemiologic studies and to adjust for measurement error in phthalate exposure.

## Materials and Methods

Eleven men from our ongoing study of the relationship between environmental agents and male reproductive health agreed to participate in the phthalate variability study. Participant recruitment into the environmental agents and male reproductive health study has been previously described (Hauser et al. 2003). Briefly, men who were the partner in couples seeking fertility evaluation for inability to conceive were recruited to participate. The study site was the Massachusetts General Hospital (MGH) Andrology Laboratory, so most men resided in the New England area. At the clinic visit, each man was asked to produce a single semen sample and to collect a single spot urine sample.

For each of the 11 men in the phthalate temporal variability study, up to nine additional spot urine samples were collected during three cycles over a 92-day period. Ten of these 11 men each contributed a total of 10 urine samples (nine for the variability study and one for the male reproductive study), whereas one of the men provided a total of seven samples (including six for the variability study). Nested within each of the three cycles were three urine samples, collected on the first 3 consecutive days of each cycle. The first cycle began upon enrollment into the phthalate temporal variability study, and urine samples were collected on days 0, 1, and 2. Cycles 2 and 3 began 30 days and 90 days after cycle 1, respectively. Therefore, the nine urine samples were collected on days 0, 1, and 2 (cycle 1); days 30, 31, and 32 (cycle 2); and days 90, 91, and 92 (cycle 3).

All the urine samples were collected in a sterile specimen cup. The urine sample on day 0 was collected at the MGH Andrology laboratory. All other samples were collected at the subject’s home and frozen before overnight shipment to the Harvard School of Public Health (HSPH) on blue ice. All urine samples were then shipped frozen on dry ice from HSPH to CDC. Eight phthalate monoesters—MBzP, MBP, MEP, MEHP, monomethyl phthalate (MMP), mono-*n*-octyl phthalate (MOP), mono-3-methyl-5-dimethylhexyl phthalate (MINP), and monocyclohexyl phthalate (MCHP)—were measured in each spot urine sample using an analytical approach developed at the CDC (Silva et al. 2003). Briefly, the determination of phthalate metabolites in urine involved enzymatic deconjugation of the glucuronidated metabolites, solid-phase extraction, separation with high-performance liquid chromatography, and detection by tandem mass spectrometry. Detection limits were in the low micrograms per liter range. Reagent blanks and ^13^C_4_-labeled internal standards were used along with conjugated internal standards to increase the precision of the measurements. One method blank, two quality control samples (human urine spiked with phthalates), and two sets of standards were analyzed along with every 21 unknown urine samples. Analysts at the CDC were blind to all information concerning subjects.

Several methods adjust for urine volume (Boeniger et al. 1993; Teass et al. 1998). Although creatinine is a frequently used form of adjustment, if a compound is excreted primarily by tubular secretion it is not appropriate to adjust for creatinine level (Teass et al. 1998). Although the methods of excretion of the phthalate monoesters measured in this study are unknown, terephthalic acid was found to be actively secreted by renal tubules and actively reabsorbed by the kidney (Tremaine and Quebbemann 1985). Furthermore, because organic compounds that are glucuronidated in the liver, like the phthalates, are eliminated by active tubular secretion (Boeniger et al. 1993), creatinine adjustment may not be appropriate for phthalates. Additionally, creatinine levels may be confounded by muscularity, physical activity, urine flow, time of day, diet, and disease states (Boeniger et al. 1993; Teass et al. 1998). For these reasons, specific gravity, rather than creatinine, was used to normalize phthalate levels.

Urinary phthalate levels were normalized for dilution by specific gravity adjustment using the formula *P_c_* = *P* × [(1.024 – 1)/(SG – 1)], where *P_c_* is the specific-gravity–corrected phthalate concentration (micrograms per liter), *P* is the observed phthalate concentration (micrograms per liter), and SG is the specific gravity of the urine sample (Boeniger et al. 1993; Teass et al. 1998). Specific gravity was measured using a handheld refractometer (National Instrument Company, Inc., Baltimore, MD), which was calibrated with deionized water before each measurement.

### Statistical analyses.

We performed the statistical analyses using the Statistical Analysis Software (SAS), version 8.1 (SAS Institute, Cary, NC). Both unadjusted and specific-gravity–adjusted values were used. For values below the limit of detection (LOD), corresponding to 1.2 (MEP), 0.94 (MBP), 0.47 (MBzP), 0.86 (MEHP), 0.70 (MMP), 0.77 (MOP), 0.79 (MINP), and 0.93 μg/L (MCHP), we used an imputed value equal to one-half the LOD.

We constructed graphs to compare metabolite levels within and between subjects, and calculated Spearman correlation coefficients to investigate correlations between samples collected at different time points.

To assess between- and within-person variability of metabolite levels, we calculated ICCs for each metabolite based on output from a random effects model fit using PROC MIXED (Rosner 1999). ICC, defined as the ratio of between-person variance to total variance, is a measure of reliability of repeated measures over time. ICC ranges from 0 to 1, with values near 1 indicating high reliability and values near 0 indicating poor reliability. ICC can also be used in an internal validity study to account for measurement error in epidemiology effect estimates (Carroll et al. 1995; Rosner et al. 1992).

To apportion variances among nested components, we fit a hierarchical model (using PROC MIXED). For a more robust estimate of between-subject variability, we used the results of the single urine samples collected from all 369 men enrolled so far in the ongoing environmental agents and male reproductive health study in the variance apportionment analysis. Because the 11 men in this variability study were also enrolled in the male reproductive health study, their single urine sample collected for the reproductive study contributed additional information on variability. Within-subject variance was further apportioned into cycle-to-cycle variance and day-to-day variance (Box et al. 1978). Day-to-day variance was defined as the variance in phthalate metabolite levels between samples 1 or 2 days apart, regardless of whether they were collected in cycle 1, 2, or 3. Cycle-to-cycle variance was defined as the variance between the three cycles minus the day-to-day variances within the cycles. Because day is nested within cycle, the cycle-to-cycle variance uses information from the three nested daily samples in cycles 1, 2, and 3.

Although ICC is an indicator of reliability for continuous measures, it does not measure the extent of exposure misclassification that may occur if exposure is categorized into tertiles of low, medium, and high exposure. To explore categorical exposure misclassification, we performed sensitivity and specificity analyses and surrogate category analyses. In both analyses, tertiles were created using the mean of the nine repeat urine samples for each of the 10 subjects in the variability study. The subject with only six repeat urine samples was not included in these analyses because he did not have complete data. Tertiles based on the 369 single urine samples from subjects in the male reproductive health study produced an unbalanced and unstable design because some of these tertiles contained zero subjects from the variability study. This led to nonidentifiable results for that tertile. Therefore, analyses using tertiles based on the 369 single urine samples are not presented.

In the surrogate category analysis, we calculated actual values for surrogate categories to show the quantitative differences in phthalate metabolite levels that correspond to the relative categories defined by a single urine sample from the 10 variability subjects (Willett 1998). We grouped variability subjects first into tertiles by treating each of the nine repeat urine samples as a single spot urine sample (i.e., the surrogate method). For instance, for each of the nine repeat urine samples, the 10 subjects were categorized into high, medium, or low tertiles. The “true value” for these same subjects based on their 3-month average phthalate metabolite levels (using all the nine replicate samples) was then assigned to the tertiles defined by the single (surrogate) sample. Each of the nine samples was used as the surrogate sample in separate calculations to check for consistency. Each subject’s 10th sample from the male reproductive health study was not used in this analysis because this sample could have been collected up to 12 months earlier.

We also evaluated sensitivity and specificity of a single urine sample as a predictor of high and low tertiles of 3-month average phthalate metabolite levels by comparing the distribution of predicted and observed levels for agreement. For observed or “true” exposure, we calculated 3-month average metabolite levels (using all the nine replicate samples) for each subject and divided the 10 subjects into tertiles. The distribution of 96 individual samples (10 subjects providing nine replicate samples, one subject providing six) was then also divided into tertiles, with each sample representing a predicted value based on a single spot urine sample. For each sample time (days 0–92), agreement between predicted and observed “true” tertile categorization was scored across all subjects, resulting in nine separate contingency tables. All nine tables were then combined into a single table, where overall sensitivity and specificity were calculated (Peck et al. 2003). The same method was used to assess the sensitivity and specificity if two samples, and then additionally if three samples were taken for each subject at least one cycle apart within a 92-day time period. When evaluating the sensitivity of two and three samples, all possible combinations of sample pairings from the nine repeated samples, excluding samples from the same cycle, were used in the analysis. The goal was to simulate and compare the ability of exposure assessments that involve one, two, or three urine samples to predict a subject’s “true” 3-month average exposure tertile classification.

## Results

We measured eight phthalate metabolites in urine. However, because > 75% of the samples had nondetectable levels of MCHP, MOP, and MINP, the results for these three metabolites were not informative and were not included in the analyses. MEP was detected in 100% of samples, whereas MBP, MBzP, MEHP, and MMP were detected in > 90% of samples.

**Figure 1:**
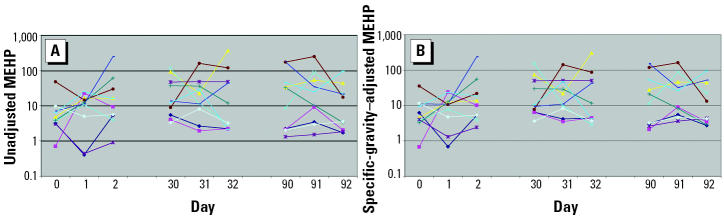
Nine repeated urine samples collected from 10 men over a 3-month period: MEHP. (*A*) Unadjusted. (*B*) Specific-gravity adjusted.

**Figure 2:**
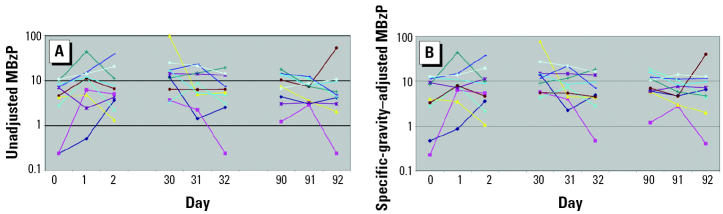
Nine repeated urine samples collected from 10 men over a 3-month period: MBzP. (*A*) Unadjusted. (*B*) Specific-gravity adjusted.

**Figure 3:**
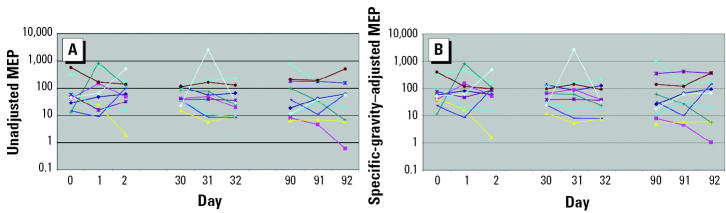
Nine repeated urine samples collected from 10 men over a 3-month period: MEP. (*A*) Unadjusted. (*B*) Specific-gravity adjusted.

**Figure 4:**
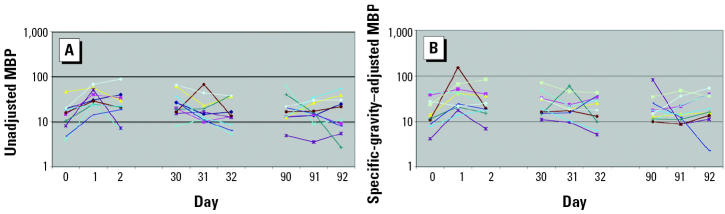
Nine repeated urine samples collected from 10 men over a 3-month period: MBP. (*A*) Unadjusted. (*B*) Specific-gravity adjusted.

**Figure 5:**
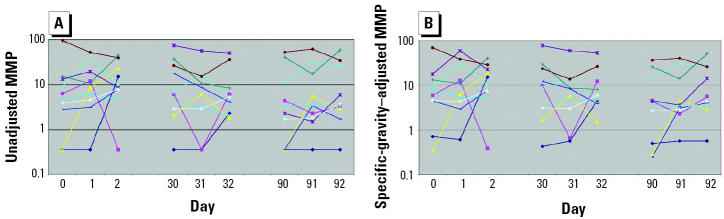
Nine repeated urine samples collected from 10 men over a 3-month period: MMP. (*A*) Unadjusted. (*B*) Specific-gravity adjusted.

Unadjusted and specific-gravity–adjusted median concentrations of MEP, MBP, MBzP, MEHP, and MMP from the 369 men who provided a single urine sample for the environmental agents and male reproductive health study are presented in Table 1. Of these 369 men, 11 also participated in the variability study. Ten of the 11 men provided nine urine samples collected over 92 days, whereas 1 man collected six urine samples over 32 days only. In Figures 1–5, the unadjusted (Figures 1A–5A) and specific-gravity–adjusted (Figures 1B–5B) urinary phthalate metabolite concentrations are plotted by day for each subject (the one subject with only six urine samples was not plotted). Even after dilution adjustment, there was still substantial variability in phthalate metabolite concentrations over time. MEHP concentrations showed large within-subject variability, whereas MEP showed less within-subject variability.

Of the total subject variance among the 369 subjects, the day-to-day variance component ranged from 27.2% (MBP) to 58.1% (MMP), whereas the cycle-to-cycle variances ranged from 1.5% (MBP) to 16.3% (MEP). Cycle-to-cycle variance is the within-subject variance remaining after day-to-day variance is calculated using the replicate samples nested within each of the three cycles. These results suggest that, after accounting for day-to-day variance, there is little additional cycle-to-cycle variance. Therefore, if we were to collect only two urine samples a day apart, we would account for 83.7–98.5%, depending on the phthalate monoester, of the total subject variance, which is composed of between- and within-subject variance. Likewise, if we collected two urine samples 1 month apart we would account for both cycle-to-cycle and day-today variability, or 100% of the within-subject variance.

To determine the predictive ability of a single urine sample to correctly categorize a subject’s exposure into high, medium, or low tertiles, we calculated actual values (mean and geometric mean values) for surrogate categories. The results are presented in Table 3 (only the 10 subjects who provided nine urine samples each were used in this analysis). Overall, the results suggest that a single spot urine sample was predictive of the 3-month average exposure because there were monotonic increasing geometric means across tertiles. For instance, for MBP, when a single sample on day 0 was used to group subjects into low-, medium-, and high-exposure groups, the “true” geometric mean MBP levels increased from 12.7 μg/L in the group designated as low exposure, to 22.8 μg/L in the medium-exposure group, to 28.3 μg/L in the high-exposure group. Although single spot urine samples were generally predictive, there were differences in the predictive ability of a single urine sample for different phthalate mono-esters. A single urine sample was least predictive for MEHP, where only five of the nine spot urine samples produced a monotonic increasing geometric mean. In contrast, eight of the nine spot urine samples produced monotonic increasing geometric means for MBzP, MBP, MEP, and MMP. As expected, MEP, with the widest range in exposure levels, showed the largest difference in geometric means between low-, medium-, and high-exposure categories.

For a more quantitative assessment of how well a single urine sample predicts a subject’s exposure category based on 3-month average metabolite levels, we conducted sensitivity and specificity analyses, using only the results from the 10 subjects who provided nine urine samples each (Table 4). The proportion of men who truly had the highest 3-month average exposure (top 33%) that would be identified as such using single urine samples anytime throughout that 3-month period (i.e., sensitivity) ranged from 0.56 for MEHP to 0.74 for MMP. The proportion of men with truly comparatively low exposure (tertiles 2 and 3) that were classified correctly (i.e., specificity) ranged from 0.83 for MEHP to 0.90 for MMP. Sensitivity analyses for one, two, or three urine samples are presented in Table 4. When two samples were collected 1–3 months apart, there were small increases in sensitivity and specificity, especially evident for MEHP. When three urine samples were collected, each 1–3 months apart, sensitivity moderately increased for MEHP and MMP, with slight increases for the other monoesters. In contrast, when three urine samples were collected on 3 consecutive days, sensitivity for MEHP, MBzP, MBP, and MMP did not increase. However, sensitivity did increase for MEP.

We also performed all analyses described above using unadjusted phthalate levels (data not shown). Overall, variance apportionment and sensitivity analyses were very similar to the specific-gravity–adjusted results shown above. The surrogate exposure category method differed slightly with less consistent dose–response categories found for the unadjusted phthalate metabolite levels.

## Discussion

Although the present study found substantial within-subject variability in urinary phthalate metabolite levels, the sensitivity of a single spot urine sample to predict 3-month average phthalate exposure was moderate to high. As expected, because phthalates are rapidly metabolized and do not bioaccumulate, the collection of additional urine samples 1–3 months apart improves the prediction of a subject’s 3-month average exposure. The levels of urinary metabolite levels found in the present study were similar to reference ranges measured in U.S. males for NHANES 1999–2000 (CDC 2003; Silva et al. 2004).

The predictive ability of a single urine sample to determine a subject’s 3-month average exposure varied across phthalates. For MEHP, a single urine sample was least predictive of the tertile categorization and had the lowest sensitivity (Table 4). This implies that in statistical analyses in which only a single urine sample is available to categorize a subject’s 3-month exposure to MEHP, there is likely exposure measure misclassification resulting in bias toward the null hypothesis for exposure–response relationships. MEHP has been associated with developmental reproductive toxicity in laboratory studies (Oishi 1986; Park et al. 2002; Sjoberg et al. 1986). However, in our previously published study, we did not find an association between MEHP and semen parameters among adult men (Duty et al. 2003). In contrast, we did find associations of MBP and MBzP with semen parameters. Although our study differed from the animal studies because we measured adult and not gestational exposure, our findings suggesting that a single urine sample, used to categorize a subject’s exposure, did not adequately measure 3-month average exposure to MEHP. This may partially explain our inability to detect associations between semen parameters with MEHP. To improve upon our exposure classification of MEHP, we are currently collecting two urine samples 1 month apart from all subjects. This will allow us to use measurement error correction methods to adjust for exposure misclassification of phthalate exposure (Carroll et al. 1995).

It is possible the calculated sensitivities and specificities may be slightly overestimated to a small degree because we included predicted values in the calculation of the observed values. Therefore, the errors of predicted and observed values are not totally independent, which can lead to an overestimation of sensitivity and specificity (Willett 1998). Similarly, a portion of the increased sensitivity and specificity observed when taking two or three samples per subject instead of a single sample may be caused partly by the increased dependence between the errors of the predicted and observed values.

Apportioning the sources of variability in urinary phthalate metabolite levels can be used to design more valid and efficient exposure assessments. As expected, the urinary phthalate metabolite concentrations in samples collected close together in time, separated by 1–2 days, were more correlated than those in samples collected farther apart in time, separated by 1–3 months. Two samples collected a month or more apart include variability in urinary phthalate metabolite levels contributed to both by day-to-day changes in exposure and by monthly trends in phthalate exposure, such as seasonal changes in diet, personal product use, or activity patterns, as well as other environmental or biologic factors.

For each health end point of interest in an epidemiologic study, the relevant time window over which exposure is measured needs to be defined. For acute responses after acute exposures, a single urine sample may be adequate to define phthalate exposure. However, we are generally interested in health end points that have exposure windows of months, if not years. To this end, accurate exposure assessment depends on a strategy whereby we accurately measure exposure over these time windows. The simplest approach is to collect multiple urine samples from all subjects over the time interval of interest. However, it is not always feasible to collect multiple urine samples because of both cost constraints and limitations imposed by the subject’s commitments to multiple collections. Based on the results of this phthalate variability study, for male reproductive health end points, we recommend collecting at least two urine samples 1–3 months apart. This will provide an estimate of the within-person variability taking into account both month-to-month and day-to-day variance. Nevertheless, if the study design only permitted collecting two samples 1–2 days apart, this, too, would provide a reasonable estimate of within-subject variance contributed to by day-to-day variance. After collection of the replicate urine samples in either sampling scheme, measurement error models could then be used to adjust for measurement error in exposure (Carroll et al. 1995). A discussion of this is beyond the scope of this report.

In conclusion, although a single urine sample was moderately predictive of 3-month exposure to phthalates, the predictive ability varied across phthalate monoesters. A single urine sample was more predictive for MEP and less predictive for MEHP. The single sample performed well in classifying a subject’s exposure into tertiles, and the amount of nondifferential random exposure misclassification is likely to be moderate or small for most phthalate metabolites of interest. The variance apportionment analysis suggests that two urine samples, the second collected 1–3 months after the first sample, is the minimum number of samples necessary to account for the within subject day-to-day and cycle-to-cycle variability in urinary phthalate metabolite levels. Because the degree of between- and within-subject variance and thus the predictive ability of a single urine sample differ among phthalate metabolites, the most efficient exposure assessment strategy for a particular study depends on the phthalates of interest. The results from the present study will be used in our ongoing environmental agents and male reproductive health study to correct for measurement error in the effect estimates of exposure–response relationships between phthalates and sperm function. The findings from this variability study may also be pertinent to other end points with relevant exposure periods of several months. However, if the study population is not adult men of reproductive age, such as studies involving children or pregnant women, we recommend that a variability study be conducted to determine population-specific exposure assessment strategies.

## Correction

The values in Tables 1 and 3 have been rounded from those in the manuscript published online to reflect laboratory sensitivities. In Table 2, for all subjects the SE for ln(MBzP) day is 0.10, and that for percent of total variance ln(MEP) day is 40.9. The errors have been corrected here.

## Figures and Tables

**Table 1:** Distribution of phthalate metabolite levels (μg/L) measured in a single spot urine sample from 369 men.

		Selected percentiles					
Phthalate metabolite	Geometric mean	10th	25th	50th	75th	90th	95th
Unadjusted							
MEHP	5.7	0.5	1.9	5.2	17.2	63.6	110
MBzP	5.6	1.1	2.4	6.0	13.7	25.3	34.7
MEP	149	22.7	46.1	128	444	1,144	1,879
MBP	13.3	2.9	7.0	13.6	29.3	50.4	73.1
MMP	3.8	0.4	1.7	4.4	9.6	21.5	29.9
Specific-gravity adjusted							
MEHP	6.8	0.8	2.4	6.5	19.5	64.5	120
MBzP	6.6	1.8	3.8	7.2	14.0	22.8	36.2
MEP	175	30.7	58.7	153	495	1,145	1,897
MBP	15.8	4.8	9.8	16	29.2	45.9	66.7
MMP	4.5	0.6	2.2	4.9	11.7	22.4	30.4

**Table 2:** Variance apportionment for specific-gravity–adjusted phthalate levels in urine.

	Variability subjects only (*N* = 11, *n* = 96)		All subjects*^a^* (*N* = 369, *n* = 465)	
	Variance estimate ± SE	Percent of total variance	Variance estimate ± SE	Percent of total variance
ln(MEHP)				
Subject*^b^*	0.49 ± 0.32	27.9	1.57 ± 0.31	54.3
Cycle*^c^*	0.30 ± 0.20	17.3	0.33 ± 0.21	11.4
Day*^d^*	0.97 ± 0.17	54.7	0.99 ± 0.18	34.3
ln(MBzP)				
Subject	0.47 ± 0.25	42.5	0.80 ± 0.14	55.1
Cycle	0.075 ± 0.086	6.9	0.083 ± 0.09	5.7
Day	0.54 ± 0.096	50.5	0.57 ± 0.10	39.2
ln(MEP)				
Subject	1.08 ± 0.59	46.2	0.87 ± 0.19	42.9
Cycle	0.33 ± 0.20	14.5	0.33 ± 0.16	16.3
Day	0.89 ± 0.16	39.3	0.83 ± 0.13	40.9
ln(MBP)				
Subject	0.14 ± 0.085	29.0	0.84 ± 0.10	71.3
Cycle	0.025 ± 0.046	5.0	0.018 ± 0.041	1.5
Day	0.33 ± 0.058	66.0	0.32 ± 0.059	27.2
ln(MMP)				
Subject	1.11 ± 0.55	51.7	0.51 ± 0.21	27.4
Cycle	0.011 ± 0.12	0.5	0.27 ± 0.25	14.5
Day	1.03 ± 0.18	47.8	1.08 ± 0.19	58.1

Abbreviations: *N*, number of subjects; *n*, number of samples.

a Includes 10 variability subjects who provided 10 samples each, 1 subject who provided 7 samples, plus 358 subjects who provided a single sample.

b Between-subject variance.

c Variance between three cycles after accounting for nested day-today variance.

d Variance between 3 consecutive days within a cycle.

**Table 3:** Values for surrogate exposure categories comparing a single urine sample with 3-month average levels based on nine replicates from 10 men.

	Mean (μg/L)			Geometric mean (μg/L)		
Surrogate	Low	Medium	High	Low	Medium	High
MEHP						
Day 0	19.9	28.9	44.7	9.3	11.5	18.3
Day 1	9.5	55.7	19.4	5.1	26.3	11.0
Day 2	18.8	26.2	49.3	7.1	11.1	25.2
Day 30	5.6	44.9	37.6	4.4	24.3	14.2
Day 31	5.6	43.8	39.0	4.4	20.1	18.4
Day 32	23.6	23.2	48.6	11.2	9.2	20.5
Day 90	10.0	29.7	53.5	5.0	12.7	29.9
Day 91	9.5	30.1	53.5	5.1	14.3	24.8
Day 92	10.2	31.1	51.5	6.1	11.7	27.5
MBzP						
Day 0	4.9	11.2	14.3	3.1	7.5	13.2
Day 1	6.6	9.3	15.2	2.9	7.8	13.3
Day 2	7.8	9.2	14.0	4.3	6.0	12.8
Day 30	6.3	11.2	12.8	3.9	8.5	8.9
Day 31	6.6	10.1	14.0	2.9	8.0	12.8
Day 32	7.2	10.1	13.4	3.5	7.5	11.9
Day 90	8.3	11.1	11.0	4.2	7.8	9.2
Day 91	8.0	9.9	12.9	3.7	7.8	10.6
Day 92	7.2	9.9	13.7	3.5	7.8	11.3
MEP						
Day 0	73.2	158.4	172	28.9	46.8	94.4
Day 1	26.6	199.6	163	15.8	98.3	64.1
Day 2	30.2	113	276	6.2	70.2	97.8
Day 30	146	92.8	186	24.4	39.7	139
Day 31	26.6	104	291	15.8	53.1	146
Day 32	26.6	182	186	15.8	55.0	139
Day 90	30.2	158	216	16.2	50.2	153
Day 91	33.4	155	216	16.0	50.7	153
Day 92	63.9	132	216	20.3	42.4	153
MBP						
Day 0	16.3	30	30.5	12.7	22.8	28.3
Day 1	16.7	24.9	37.3	13.1	21.6	29.3
Day 2	16.7	26.3	35.5	13.1	20.5	31.4
Day 30	18.0	23.4	38.1	13.5	18.6	34.7
Day 31	16.3	27.7	34.0	12.7	22.7	28.5
Day 32	16.7	27.7	33.6	13.1	22.7	27.6
Day 90	26.4	24.4	28.4	19.8	21.6	19.4
Day 91	21.7	22.0	36.3	14.4	18.7	32.3
Day 92	21.3	26.6	30.5	13.9	21.3	28.3
MMP						
Day 0	4.2	7.9	30.2	2.0	5.6	23.2
Day 1	4.0	9.4	28.3	2.4	5.2	21.3
Day 2	5.0	7.3	30.2	4.1	3.3	23.2
Day 30	3.9	8.1	30.2	2.1	5.3	23.2
Day 31	4.3	9.2	28.3	2.5	5.0	21.3
Day 32	4.9	11.5	24.8	3.3	5.7	13.7
Day 90	4.2	12.4	24.3	2.0	6.2	19.9
Day 91	4.3	12.3	24.3	2.5	5.3	19.9
Day 92	4.0	12.5	24.3	2.2	5.8	19.9

Only samples from the 10 subjects who provided nine urine samples each were used in this analysis.

**Table 4:** Sensitivity and specificity for predicting men with the highest 3-month average phthalate levels (top 33%) with one, two, or three urine samples (*n* = 10 men, 90 samples).

	MEHP		MBzP		MEP		MBP		MMP	
No. of samples	Sensitivity	Specificity	Sensitivity	Specificity	Sensitivity	Specificity	Sensitivity	Specificity	Sensitivity	Specificity
One sample	0.56	0.83	0.63	0.84	0.63 (0.67)*^a^*	0.87 (0.86)	0.67	0.87	0.74	0.90
Two samples (at least 1 month apart)	0.63	0.84	0.65	0.85	0.69 (0.78)	0.88 (0.90)	0.65	0.85	0.81	0.92
Three samples (at least 1 month apart)	0.73	0.88	0.69	0.87	0.68 (0.70)	0.88 (0.87)	0.68	0.86	0.90	0.96
Three samples (3 consecutive days)	0.56	0.81	0.67	0.86	0.78 (0.67)	0.90 (0.90)	0.67	0.86	0.78	0.90

Only samples from the 10 subjects who provided nine urine samples each were used in this analysis.

a Values in parentheses are sensitivity and specificity using geometric mean ranks instead of arithmetic mean ranks for observed tertile classification; for the other four phthalates these values were identical.
